# A Word with Our Readers

**Published:** 1872-04

**Authors:** 


### A WORD WITH OUR READERS.

We wish to state, emphatically, for the purpose of correcting any misapprehension on the part of our readers, that our Associate and Corresponding Editors are not responsible, *in any sense*, for any article which may appear in, or for the general management of, THE COMPANION. This we state as a matter of justice to them—otherwise some unthinking and hypercritical individual might so regard them. They are responsible *alone* for what they write, and not for *our* articles or opinions.

